# Dosing-Time Dependent Effects of Sodium Nitroprusside on Cerebral, Renal, and Hepatic Catalase Activity in Mice

**DOI:** 10.1155/2015/790480

**Published:** 2015-03-15

**Authors:** Mamane Sani, Hichem Sebai, Roberto Refinetti, Mohan Mondal, Néziha Ghanem-Boughanmi, Naceur A. Boughattas, Mossadok Ben-Attia

**Affiliations:** ^1^UMR Biosurveillance et Toxicologie Environnementale, Département de Biologie, Faculté des Sciences et Techniques de Maradi, 465 Maradi, Niger; ^2^Circadian Rhythm Laboratory, Boise State University, 1910 University Drive, Boise, ID 83725, USA; ^3^UR Ethnobotanie et Stress Oxydant, Département des Sciences de la Vie, Faculté des Sciences de Bizerte, 7021 Zarzouna, Tunisia; ^4^National Dairy Research Institute, Eastern Regional Station, A-12, Kalyani,West Bengal 741235, India; ^5^Laboratoire de Pharmacologie, Faculté de Médecine, 5019 Monastir, Tunisia; ^6^Laboratoire de Biosurveillance de l'Environnement, Faculté des Sciences de Bizerte, 7021 Zarzouna, Tunisia

## Abstract

To investigate the time dependence of sodium nitroprusside- (NPS-) induced oxidative effects, the authors study the variation of the antioxidant enzyme CAT activity in various tissues after the administration of a single 2.5 mg/kg dose of SNP or sodium chloride (NaCl 0.9%). For each of the two dosing times (1 and 13 hours after light onset, HALO, which correspond to the beginning of diurnal rest span and of nocturnal activity span of mice, resp.), brain, kidney, and liver tissues were excised from animals at 0, 1, 3, 6, 9, 12, 24, and 36 h following the drug administration and CAT activity was assayed. The results suggest that SNP-induced stimulation of CAT activity is greater in all three tissues when the drug is administered at 1 HALO than at 13 HALO. Two-way ANOVA revealed that CAT activity significantly (*P* < 0.004) varied as a function of the sampling time but not of the treatment in all three tissues. Moreover, a statistically significant (*P* < 0.004) interaction between the organ sampling-time and the SNP treatment was revealed in kidney regardless of the dosing time, whereas a highly significant (*P* < 0.0002) interaction was validated in liver only in animals injected at 13 HALO.

## 1. Introduction

The use of SNP as an antihypertensive agent [1–4] in a growing list of clinical conditions has been associated with cyanide- (CN^−^-) induced toxicity [5, 6]. Moreover, previous reports revealed that besides these released CN^−^ ions, other metabolites as nitric monoxide (NO) [7, 8] may also contribute to the toxicity of this drug trough generation of reactive oxygen species (ROS) such as superoxide ion (O_2_^∙−^) [5] and hydrogen peroxide (H_2_O_2_) [9]. As for many other drugs, side toxic effects of SNP have been reported both in experimental [10–13] and in clinical [14, 15] designs. Indeed, it has been reported that, within minutes of infusion, SNP decomposes into metabolites that are pharmacologically inactive but toxicologically important [16]. Thus, one molecule of SNP is metabolized by combination with haemoglobin to produce one molecule of cyanmethaemoglobin and four CN ions [17]. Despite this, there have been few reported cases of CN^−^ toxicity following the therapeutic administration of SNP [18–20]. It is well established that the toxic free CN^−^ can be converted* in vivo* into the much less toxic thiocyanate (SCN) by a ubiquitous enzyme rhodanese [21] that is present in various tissues [22–25] of all living organisms, from bacteria to humans [26–28]. SNP-induced oxidative damage has also been reported [13, 29]. This phenomenon occurs when there is an impairment of the balance between pro- and antioxidant systems. It is well known that SNP-induced oxidative effects are related to the release of NO that might be potentially toxic [7, 8]. Previous studies reported that high levels of NO may interact with ROS or other molecules to generate many more ROS that may induce lipid peroxidation (LPO) in various tissues [30, 31], including brain, kidney, and liver. Moreover, organisms have a wide spectrum of mechanisms to defend against the potential oxidative damage from ROS formation, including antioxidant molecules that directly inactivate ROS and enzymes that metabolically convert toxic compounds to forms that are readily excreted. Among these antioxidant enzymes, CAT seems to play an important role by catalyzing the rapid degradation [32, 33] of H_2_O_2_ to water (H_2_O) and oxygen (O_2_). Since there is previous evidence that ROS are important in initiating several pathophysiological processes, especially ischemic reperfusion injury [34, 35], the role of antioxidant enzymes in health and disease is under increasing investigation. Because much of this damage occurs within cells, the antioxidant activity in tissue is considered to be more relevant than that in plasma [36]. Thus, deficiencies compromising the capacity to detoxify oxidant molecules such as H_2_O_2_ and O_2_ radicals result in oxidant-induced denaturation of intracellular molecules and premature destruction of target cells. Other enzymopathies that might compromise intracellular reductive capacity have also been described; they include abnormalities involving glutathione peroxidase (GPx) and glutathione reductase (GSR) activity [37, 38]. Therefore, any reduced capacity to deal with oxidative stress might involve diminished activity in various antioxidant enzymes or reflect a diminished reserve in the reductive capacity in mutant cells. Although it is clear that antioxidants play an important role in protecting cells, knowledge of molecular mechanisms that regulate their expression is limited. Expression of various genes encoding antioxidant enzymes is partly mediated by a* cis*-active DNA element designated the antioxidant response element (ARE) [39]. Thus, it is believed that the increase in cell sensitivity results from decreased expression of oxidative-stress response genes. Moreover, the report on downregulation of glutathione-S-transferase (GST) and CAT genes in mutant cells provides a plausible explanation for a molecular basis of the observed sensitivity to oxidant compounds [40]. Since it has been previously reported that CAT plays an essential role in the detoxification of H_2_O_2_-derived radical species in cells [41–43], a reduction in that enzyme's activity would be an important factor in the sensitivity of tissues to oxidative stress. Nevertheless, it is likely that expression of a broad set of other enzymes is also affected. Other authors revealed that low concentrations of SNP had a prominent effect in producing oxidative damage in platelets in comparison to rat brain tissue [44]. Similarly, we recently revealed that a neurotoxic 2.5 mg/kg dose of SNP induced LPO in brain, kidney, and liver tissues [13], but not in erythrocytes [45]. Therefore, any variation of oxidative damage among organs might be related to their respective sensitivity and/or antioxidant system efficiency. For that and since we previously found that these three tissues showed different sensitivity to SNP-induced LPO [13], the potentiality and efficiency of their antioxidant systems deserve to be investigated. Hence, we were interested in examining the effects of SNP, a well-known NO donor, on an antioxidant enzyme CAT activity. Since SNP also varies in potency and/or toxicity according to the administration time [11–13, 45], the present study describes the variation of CAT activity in mouse brain, kidney, and liver after* i.p.* administration of a 2.5 mg/kg dose at two circadian times (1 and 13 HALO).

## 2. Materials and Methods

### 2.1. Animals and Housing

Male* Swiss albino* mice (≈25 g body weight) aged 7–9 weeks (SIPHAT, 2013 Foundouk-Choucha, Tunisia) were used. They were acclimated for at least 3 weeks prior to and during experiments [46] in two-air conditioned rooms specially designed for chronobiological investigations by having an inverted light regimen to explore several circadian stages during the usual diurnal work span. In one room, the lights were on from 07:00 to 19:00 h; in the other room, the lights were on from 19:00 to 07:00 h (D : L 12 : 12; reversed lighting regimen). Thus, animals were synchronized with an alternating 12 h light (L)/12 h dark (D) cycle. The room temperature was maintained at 22 ± 2°C and the relative humidity was about 50–60%. During all experiments, a standard diet (Purina Rat Chow; SICO, Sfax 3000, Tunisia) and water were provided* ad libitum*. Animals were randomly divided into four different and comparable groups of 48 mice each at one of the two dosing times 1 and 13 HALO (Table 1). All experiments were performed according to the guidelines of care and use of laboratory animals.

### 2.2. Drug and Animal Treatment

SNP was kindly supplied by the National Laboratory of Drug Control (1006 Tunis, Tunisia) in the hydrosoluble form (Na_2_[Fe(CN)_5_NO]·2H_2_O). Reagents KH_2_PO_4_, K_2_HPO_4_, and H_2_O_2_ (110 Vol) stock solutions were obtained from Sigma Aldrich s.r.l. (Milano, Italy) and Merck (Darmstadt, Germany) and were of the highest commercial grade available. Substrate solutions were prepared with distilled water immediately before use. Based on our previous experience with SNP in chronotoxicological studies, in adult mice, neurotoxic effects of SNP were triggered with doses ranging from 2.5 to 5 mg/kg-a median toxic dose TD_50_ (dose inducing 50% motor-inco-ordination) equal to 3.6 ± 0.5 mg/kg. Since oxidative effects of SNP seem to be related to its neurotoxicity, the lowest neurotoxic SNP dose (of 2.5 mg/kg) was used to investigate SNP-induced oxidative effects.

SNP was freshly prepared on each dosing time of the study by adding an adequate volume of sterile distilled water to obtain the desired concentration. For animal experiments, the particular recommendations and approval of protocols were obtained. A single 2.5 mg/kg dose of SNP was administrated to mice by* i.p.* route in a fixed fluid volume (10 mL/kg b.w.) at each of the two circadian times (1 and 13 HALO). Each circadian time involved different but comparable subgroups of mice (*n* = 6) corresponding to animals sacrificed by decapitation at 0, 1, 3, 6, 9, 12, 24, and 36 h after injection. Thereafter, brain, kidney, and liver tissues were quickly removed and individually categorized with respect to tissue, dosing-time, and sampling-time and then stored frozen at −84°C until assayed.

### 2.3. Assay Procedure of CAT Activity

For each animal, whole brain, kidneys, and a portion of liver were separately homogenized (5%, w/v) in 20 vol. cold phosphate buffer (10 mM KH_2_PO_4_–10 mM K_2_HPO_4_, pH 7.0) using a Petri dish maintained in finely crushed ice. CAT activity was determined at room temperature (26–28°C) as described by Claiborne [33]. This method was previously used by the authors to explore temporal variations of CAT activity in brain, kidney, and liver of mice in nonstress conditions [47]. Prior to the measurement of enzyme activity, we demonstrated that the enzyme followed accepted chemical principles by determining the rates of enzyme activity. The rate (*R* = 1/time) of CAT activity corresponds to the inverse of length of time (in minutes) for O_2_ gas formation (visibly apparent as bubbling) after H_2_O_2_ decomposition. We noticed a linear relationship between activity and enzyme concentration. However, according to day, the CAT activity went linearly down by a very small amount. The reaction mixtures (1.95 mL) consisted of 10 mM solution buffer, 100 mM H_2_O_2_, and a sample. The reaction was initiated by the addition of H_2_O_2_ and absorbance changes were measured at 240 nm. One unit of CAT activity is defined as the amount of enzyme that decomposes 1 mmol H_2_O_2_ per minute. All the results were given as *μ*mol H_2_O_2_/min/g tissue. In addition, we assessed the precision of measurements by repeated assay of pools of homogenates that correspond to each tissue. The mean imprecision (coefficient of variation: CV) for within-sample repeatability (intra-assay, *n* = 4) and sample-to-sample reproducibility (interassay, *n* = 6) was calculated to test whether the variance or the relative SD (CV) was relatively constant between the three studied tissues. The mean CV within-assay was 6.9%, 7.1%, and 8.1% for liver, kidney, and brain, respectively. The mean CV among-assay (inter-assay) was 13.8% for liver, 14.3% for kidney, and 15.2% for brain.

### 2.4. Statistical Analysis

In the current study, mean and standard error of the mean (S.E.M.) were computed for each sampling-time-point and pertinent histograms were drawn for each circadian dosing-time. The Unpaired Student's* t*-test (InStat for MacIntosh, GraphPad Software, San Diego, CA, USA) was used to compare treatment and control groups at designated sampling-times. Two-way analysis of variance (ANOVA) was used to test the significance of differences in CAT activity from one sampling-time to the next by examining the interaction between sampling-time and treatment on the activity levels. Difference is considered statistically significant with a *P* value of <0.05.

## 3. Results

**Figure 1:**
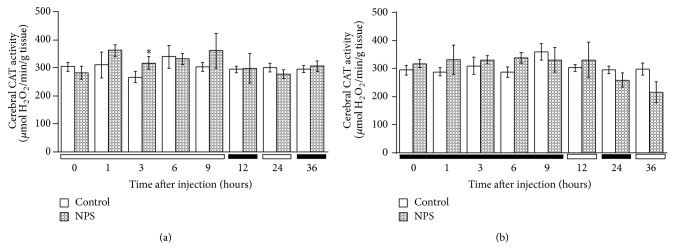
Time course of CAT activity in the brain of mice treated by* i.p.* route with SNP (2.5 mg/kg) or NaCl (0.9%). (a) Mice were treated with SNP or NaCl at 1 HALO. (b) Mice were treated with SNP or NaCl at 13 HALO. Data are expressed as mean ± SEM values from six different experiments in quadruplicate. The black bar corresponds to the dark period. Unpaired Student's* t*-test revealed statistically significance: ^*^ *P* < 0.01. Two-way ANOVA: time of sampling ((a) *F*_0.05(7,88)_ = 4.8; *P* < 0.004; (b) *F*_0.05(7,88)_ = 5.9; *P* < 0.002); treatment ((a) NS; (b) NS); time-treatment interaction ((a) NS; (b) NS).

**Figure 2:**
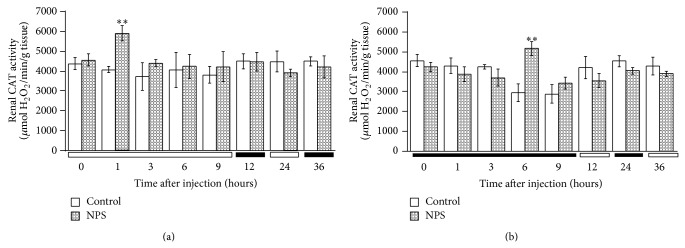
Time course of CAT activity in the kidney of mice treated by* i.p.* route with SNP (2.5 mg/kg) or NaCl (0.9%). (a) Mice were treated with SNP or NaCl at 1 HALO. (b) Mice were treated with SNP or NaCl at 13 HALO. Data are expressed as mean ± SEM values from six different experiments in quadruplicate. The black bar corresponds to the dark period. Unpaired Student's* t*-test revealed statistically significance: ^**^ *P* < 0.001. Two-way ANOVA: time of sampling ((a) *F*_0.05(7,88)_ = 14.3; *P* < 0.0001; (b) *F*_0.05(7,88)_ = 4.8; *P* < 0.003); treatment ((a) NS; (b) NS); time-treatment interaction ((a) *F*_0.05(7,88)_ = 4.3; *P* < 0.004; (b) *F*_0.05(7,88)_ = 12.7; *P* < 0.0002).

**Figure 3:**
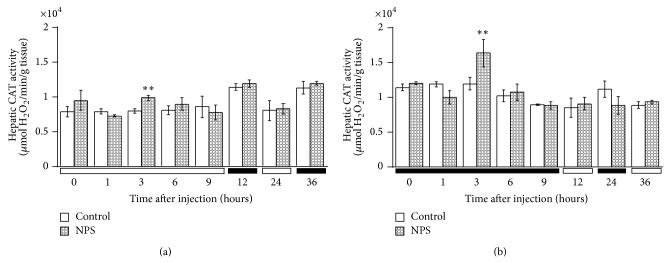
Time course of CAT activity in the liver of mice treated by* i.p.* route with SNP (2.5 mg/kg) or NaCl (0.9%). (a) Mice were treated with SNP or NaCl at 1 HALO. (b) Mice were treated with SNP or NaCl at 13 HALO. Data are expressed as mean ± SEM values from six different experiments in quadruplicate. The black bar corresponds to the dark period. Unpaired Student* t*-test revealed statistically significance: ^**^ *P* < 0.001. Two-way ANOVA: time of sampling ((a) *F*_0.05(7,88)_ = 11.8; *P* < 0.0001; (b) *F*_0.05(7,88)_ = 10.9; *P* < 0.0001); treatment ((a) NS; (b) NS); time-treatment interaction ((a) NS; (b) *F*_0.05(7,88)_ = 8.0; *P* < 0.0002).

The levels of CAT activity in relation to different sampling times are illustrated in Figures 1, 2, and 3. CAT activity significantly varied according to the sampling-time in all three tissues regardless of injection time (Tables 2 and 3). Brain CAT activity significantly (Student's* t*-test, *P* < 0.01) increased 3 h following the SNP (2.5 mg/kg,* i.p.*) administration at 1 HALO whereas the enzyme activity remained stable for the group injected at 13 HALO, regardless of sampling time (Figure 1). There was increased renal CAT activity 1 h after the SNP administration at 1 HALO (*P* < 0.001), whereas the drug significantly (*P* < 0.001) increased renal CAT activity 6 h after injection at 13 HALO (Figure 2). Hepatic CAT activity showed a statistically significant (*P* < 0.001) increase at 3 h after* i.p.* dosing regardless of the time of SNP injection (Figure 3). Two-way repeated measures ANOVA showed that CAT activity varied as a function of organ sampling-time (*P* < 0.003) but not of drug treatment (Tables 2 and 3). Nonetheless, a significant (Tables 2 and 3) interaction was detected between sampling-time and treatment in kidney and liver tissues. This interaction was not significant in brain tissue (Tables 2 and 3).

## 4. Discussion

The toxic side effects of antihypertensive drugs, including SNP, may be multifaced and various. These potential drawbacks that might evolve from the loss of cell viability due to the oxidative injury may exhibit marked variation with regard to biological administration time [11–13, 45, 48, 49]. The data presented here clearly showed that SNP administration increased the activity of the antioxidant enzyme CAT in a time-dependent manner in all studied tissues. Moreover, SNP-induced oxidative damage has been reported in various experimental [13, 45, 50] and clinical settings [51]. Paul and Ekambaram [10] reported that the administration of similar 2.5 mg/kg dose of SNP used in our study induced oxidative stress by increasing NO concentration in brain tissue. Several other experimental studies on NO-induced oxidative injury showed a dependence on both cellular redox status and cell-type specificity [13, 45, 52]. The redox status is controlled by a wide spectrum of intracellular antioxidant systems, with CAT strongly contributing to reduce ROS levels by scavenging H_2_O_2_ that is considered as the main ROS. Our present findings, which are in agreement with our previous studies [13], confirmed the oxidative effects of SNP as evidenced by the observed increase of CAT activity in all tissues studied here. The present study showed the importance of SNP administration time on the activity of the antioxidant enzyme CAT. Our results showed that CAT activity, after a single dose administration of SNP in mice, was stimulated the most when the drug was dosed at 1 than at 13 HALO. The enzyme activity significantly increased at 3 h for brain, 1 h for kidney, and 3 h for liver after the drug administration at 1 HALO. This parameter remained unchanged for brain but significantly increased at 6 h for kidney and 3 h for liver after the SNP administration at 13 HALO. These observations suggest that NO may be one of the critical factors in regulating cellular redox in conditions that are associated with the production of NO. However, the mechanism of regulation of antioxidant enzymes by NO is not yet well established. Few* in vitro* studies reported inhibitory effects of NO on antioxidant enzymes. According to Asahi et al. [53], NO has no effect on CAT and superoxide dismutase (CuZn-SOD and Mn-SOD) but only inactivates GPx by modifying the cysteine-like essential residues. On the other hand, in another study by Brown [54], NO was found to bind CAT competing with H_2_O_2_, causing a change in the optical absorbance spectrum of the heme group followed by the degradation of the enzyme. Peroxynitrite-mediated nitration on tyrosine residues and subsequent degradation of SOD has also been reported by Ischiropoulos et al. [55]. The results presented in this study clearly indicate that ROS, generated through the release of NO by SNP, increase the activity of CAT in tissues, indicating the upregulation of expression of CAT mRNA. These findings match other scientific reports that suggest an important role of CAT in preventing the reduction of cell viability induced by SNP [9]. However, Lawler and Song [56] have revealed the inhibition of CAT activity by SNP in skeletal muscle. These authors showed that the use of a 500 *μ*M SNP dose resulted in significantly lower activities for SOD, CAT, and GPx. Furthermore, other previous studies revealed that SNP-induced toxicity varies in a dose-dependent fashion and suggested that low drug doses are more cytotoxic [9]. Therefore, one hypothesis that could explain this difference is that the dose of SNP (2.5 mg/kg/10 mL ≈ 840 *μ*M) used in our investigation is higher than that used by these authors. Indeed, the use of low dose may induce the high inhibition of CAT activity with partial reversibility, probably suggesting an allosteric-like modification of the hem group of enzyme [57, 58]. It has also been reported that the increase of activities of some antioxidant enzymes by antihypertensive drugs enalapril and captopril might protect cells against oxidative damage [59]. Thus, all two-drug treatments increase SOD and Gpx activities, but not the CAT one. However, intracellular events that lead to the modulation of mRNA of antioxidant enzymes by NO or its metabolites are not known at the present time. On the other hand, reports on the antihypertensive drugs hydralazine and terazosin indicated any direct inhibitor or activator effect on antioxidant enzyme activities [60]. Moreover, these drugs induced the variation in transcriptional gene levels and modulation of posttranscriptional process of enzymes. Other findings also revealed that hydralazine or terazosin treatment significantly induce changes in the levels of mRNA for SOD, CAT, and Gpx in cardiomyocytes [61]. However, the same studies have shown that captopril decreases the level of mRNA for hepatic SOD, but not of CAT mRNA. Moreover, two-way ANOVA revealed a statistically significant interaction between sampling-time and treatment on renal CAT activity regardless of the dosing time, suggesting the influence of treatment on sampling-time-dependent variations of the enzyme activity in this tissue. However, this interaction observed only in the liver of animals injected at 13 HALO was not detected in brain tissue. It seems that the treatment-related difference was reduced in brain compared to the kidney and liver tissues. This difference in response to SNP-induced effects between the three tissues may probably be related to their respective capacities of enzymatic detoxification. Indeed, liver and kidney are two metabolically active tissues and sites of xenobiotic detoxification and, therefore, considered to be ROS powerful generators [62]. Thus, the relatively high level of CAT activities observed in these organs [47] is a determinant factor for ROS elimination, and the tissue type should be a factor that affects their efficiency and efficacy. Our previous findings showed that this enzymatic detoxification does not correlate with these damages in nonstress conditions [47, 63]. However, the absence of temporal relation between rhythmic patterns of CAT activity and chrono-oxidative effects of SNP does not mean that causal relation is not involved [64]. New investigations are needed to validate the latter and explore the contribution of other antioxidants. Nevertheless, the low level of MDA observed during the dark span in the liver [13, 63] might be correlated with the high level of CAT activity during this time [47] (see also Figure 3), suggesting the importance of this enzyme in liver. However, the oxidative injuries induced by SNP (a NO donor) were variously observed according to the type of target cells. In nucleated cells, NO may further be effective through nitrosylation of enzymes necessary for induction of cell membrane scrambling [65–69]. In our previous studies, increases of LPO induced by SNP in mouse brain, kidney, and liver tissues [13] were not evident in erythrocytes [45], suggesting that NO released from SNP is not able to cross the erythrocyte membrane to cause oxidative alterations. These results demonstrate that nucleated cells (from brain, kidney, and liver tissues) might be more sensitive to SNP-induced LPO as compared to unnucleated erythrocytes. Thus, since these different cells were exposed to SNP at similar conditions, any difference in MDA levels is expected to be a consequence of different sensitivity of tissue types. Our data clearly show that an increase in the CAT activity by SNP might be due to the overproduction of ROS as previously evidenced by the observed oxidative damage in these tissues [13]. The increase of activity of this antioxidant enzyme suggests its protective effect by the degradation of H_2_O_2_ to prevent hydroxyl (HO°) generation in these tissues.

In summary, the investigations carried out in the present study provide new data to add to the study of SNP-induced oxidative stress with reference to administration time and type of tissue, which appeared to be closely correlated to that of circadian variation in toxicity of SNP.

## Figures and Tables

**Table 1:** Main characteristics of the study investigating chronotoxicity of SNP in male *Swiss albino* mice.

Drugs	Doses	Number of mice	Dosing-time (HALO)	Toxicity variable	Time-of-sampling (HALO)
NaCl solution (control)	0.9%	48	1	CAT	0, 1, 3, 6, 9, 12, 24, 36
SNP	2.5 mg/kg	48			

NaCl solution (control)	0.9%	48	13	CAT	0, 1, 3, 6, 9, 12, 24, 36
SNP	2.5 mg/kg	48			

**Table 2:** Two-way ANOVA analyses of CAT activity variations in mouse brain, kidney, and liver after acute NPS (2.5 mg/kg) injection at 1 HALO.

Source of variance	DF	Brain		Kidney		Liver	
		*F*	*P*	*F*	*P*	*F*	*P*
Time	7	4.8	<0.004	14.3	<0.0001	11.8	<0.0001
Treatment	1	10.4	NS	0.1	NS	1.2	NS
Interaction	7	10.7	NS	4.3	<0.004	2.0	NS

ANOVA: analysis of variance.

NS: not significant (*P* > 0.05).

**Table 3:** Two-way ANOVA analyses of CAT activity variations in mouse brain, kidney, and liver after acute NPS (2.5 mg/kg) injection at 13 HALO.

Source of variance	DF	Brain		Kidney		Liver	
		*F*	*P*	*F*	*P*	*F*	*P*
Time	7	5.9	<0.002	4.8	<0.003	10.9	<0.0001
Treatment	1	10.9	NS	0.2	NS	8.2	NS
Interaction	7	11.0	NS	12.7	<0.0002	8.0	<0.0002

ANOVA: analysis of variance.

NS: not significant (*P* > 0.05).
